# Risk factors and postoperative comfort nursing in adolescents with flatfoot complicated by idiopathic scoliosis

**DOI:** 10.3389/fped.2025.1696612

**Published:** 2025-12-03

**Authors:** Suzhen Jiang, Meiya Lin, Xiaofang Wang

**Affiliations:** 1 Department of Rehabilitation, Rehabilitation Hospital Affiliated to Fujian University of Traditional Chinese Medicine, Fuzhou, China; 2 Fujian Key Laboratory of Rehabilitation Technology, Fuzhou, China

**Keywords:** adolescent idiopathic scoliosis, anxiety, blood vessels, comfort nursing, depression, flatfoot, nausea and vomiting, risk factors

## Abstract

**Objective:**

Adolescent flatfoot complicated by idiopathic scoliosis (AIS) are common musculoskeletal disorders, yet their shared risk factors and postoperative management remain underexplored. Current study aims to investigate podiatric risk factors for AIS in flatfoot patients and evaluates the efficacy of comfort nursing on postoperative nausea and vomiting following corrective surgery.

**Method:**

A two-phase study was conducted. First, 193 flatfoot patients were categorized into AIS (*n* = 144) and non-AIS (*n* = 49) groups to analyze risk factors via logistic regression. Second, the AIS patients undergoing PCS were randomized into a control group (routine care, *n* = 72) and a comfort nursing (CN) group (*n* = 72). The incidence, frequency, and severity of PONV, vital signs, medication use, and recovery outcomes were compared.

**Results:**

Age, hallux valgus angle asymmetry (*Δ*HVA), and Meary's angle asymmetry (*Δ*MA) were identified as independent risk factors for AIS in flatfoot patients. The CN group exhibited a significantly lower incidence of PONV [13/72 (18.06%) vs. 35/72 (48.61%)] and lower frequency of episodes compared to the control group (*P* < 0.001). The CN intervention also resulted in significantly reduced pain VAS scores, improved vital signs (temperature and urine output), less severe PONV-related symptoms, decreased antiemetic use, and enhanced overall recovery and patient satisfaction (all *P* < 0.05).

**Conclusion:**

Asymmetric foot morphology (*Δ*HVA and *Δ*MA) is a significant risk factor for AIS in adolescents with flatfoot. Implementing a multidimensional comfort nursing protocol effectively mitigates PONV, alleviates discomfort, and promotes postoperative recovery after PCS, suggesting that this approach could represent a valuable strategy for improving clinical outcomes in similar settings.

## Introduction

Flatfoot, a common foot deformity, is characterized by the reduction or absence of the medial longitudinal arch, with or without forefoot abduction and calcaneal valgus (1). Studies have shown that the incidence of flatfoot in children is 56.1%, declining with age, and that bilateral involvement is more common than unilateral, with a higher prevalence in males than females (2). Adolescent idiopathic scoliosis (AIS), a three-dimensional spinal deformity with a Cobb angle exceeding 10°, typically manifests during adolescence or around puberty, with an incidence ranging from 1.0% to 5.3%, higher in females than males (3, 4). AIS is characterized by spinal curvature and deformity, significantly impacting patients’ appearance and physical function (5). This condition often initiates in childhood and adolescence but can also develop in adults (6). Scoliosis results in bodily asymmetry, such as uneven shoulder and hip heights, leading to notable changes in appearance (7). Beyond aesthetic alterations, AIS can induce a range of physical issues, including back muscle imbalance and tension, causing back pain and stiffness (8). In severe cases, scoliosis can affect internal organ function, leading to respiratory difficulties, heart compression, and other complications (9).

The etiologies of both conditions are multifactorial. The foot serves as the foundational base of the body's biomechanical chain, intimately linked to the kinematics of the lower limbs, pelvis, and ultimately the spine (10). The etiology of AIS is complex, involving genetic, hormonal, environmental, and biomechanical factors (11). The foot, as a crucial element in biomechanics, is intimately linked to the lower limbs, pelvis, and spine, fulfilling essential supportive and dynamic mechanical structural functions (10). Flatfoot, with its morphological abnormalities, not only causes foot fatigue and soreness but also triggers alterations in human biomechanics (12). Patients with flatfoot are more prone to pain or discomfort in the hips, knees, and back, accompanied by increased asymmetry in knee and hip joint torques, as well as enhanced pelvic rotation and tilt (13). While AIS-related biomechanical studies have examined paraspinal muscle asymmetry, vertebral asymmetric growth, intervertebral disc wedging, and asymmetric hip and foot pressure, few have focused on the symmetry of bilateral foot morphology in AIS patients. Studies have reported decreased foot arch levels in AIS patients as scoliosis severity increases, with flatfoot identified as an independent risk factor for AIS. However, a critical gap exists in understanding which specific podiatric parameters, particularly measures of bilateral asymmetry (e.g., differences in hallux valgus angle or Meary's angle between feet), serve as key risk factors for the co-occurrence of AIS in an adolescent flatfoot population. Furthermore, there is a paucity of research on the foot parameters of patients with flatfoot concurrent with AIS (14).

The treatment of AIS varies based on individual circumstances (15). Early diagnosis and treatment are crucial for preventing further scoliosis progression in children and adolescents. In severe cases, surgical correction may be necessary (16). Posterior orthopedic surgery has emerged as a primary treatment modality for AIS, involving the insertion of screws, rods, and other devices to achieve three-dimensional spinal correction, restoring normal physiological curves and morphology (8). Surgeons devise personalized surgical plans based on patients’ specific conditions (17). Through precise surgical manipulations, effective scoliosis correction can be achieved, improving patients’ appearance and physiological functions (18). However, despite the significant achievements of posterior orthopedic surgery in AIS treatment, postoperative nausea and vomiting (P-NV) remains a non-negligible complication (19). Clinical reports indicate that P-NV is a common postoperative complication in orthopedic surgery, with varying incidence rates but generally high (20). P-NV not only causes physical discomfort and distress but can also lead to severe complications. Firstly, nausea and vomiting can induce electrolyte imbalances, particularly hypokalemia and hyponatremia, affecting patients’ normal physiological functions (21). Secondly, persistent vomiting may lead to dehydration, further burdening the body. Moreover, P-NV can impair appetite and nutrient intake, resulting in malnutrition and hindering recovery (22). Beyond the patient's well-being, P-NV imposes additional burdens on medical resources and patient families (23). Prolonged hospitalization and rehabilitation due to P-NV increase medical expenses and economic pressures on families (23). Furthermore, P-NV can negatively impact patients’ mental states, exacerbating anxiety and depression, further compromising recovery outcomes and quality of life (24).

Therefore, preventing and reducing P-NV during posterior orthopedic surgery for AIS is paramount. Surgeons must thoroughly assess patients’ risk factors preoperatively, devise individualized preventive measures, perform delicate surgeries to minimize stimulation and injury, and intensify postoperative care and monitoring to promptly identify and address P-NV and other complications (25). Only by doing so can we maximize surgical outcomes and rehabilitation quality (26). To address this issue, comfort nursing, a comprehensive nursing model emphasizing patients’ individual needs and comprehensive care measures, has been proposed (27). It encompasses strategies such as psychological support, dietary management, environmental optimization, and complementary therapies (e.g., acupressure). In P-NV prevention, comfort nursing can improve postoperative environments, provide appropriate sedation and analgesia, adjust diets and medications, and reduce N&V occurrences. While potentially beneficial, the efficacy of a structured, multidimensional comfort nursing protocol specifically targeting PONV in adolescents undergoing major spinal correction for AIS complicated by flatfoot has not been rigorously investigated.

Given the above considerations, this study aims to explore the risk factors for AIS in flatfoot patients and investigate the effect of comfort nursing on P-NV in flatfoot patients with AIS undergoing PCS. This research endeavors to enhance patients’ surgical recovery, quality of life, reduce complications, and alleviate medical expenses and burdens. Furthermore, by analyzing the preventive effects of comfort nursing on P-NV, we aim to provide effective nursing strategies, improve surgical recovery and quality of life, and offer scientific evidence and guidance for clinical practice. This study is the first to identify risk factors for AIS in adolescents. In addition, this study is the first to comprehensively evaluate the use of multidimensional comfort care in the post-AIS population. These innovations bridge gaps in existing research, emphasizing tailored interventions and multidisciplinary approaches to improve outcomes in complex orthopedic conditions.

## Methods

### Clinical data

This two-phase, prospective cohort study embedded with a randomized controlled trial was conducted at the Rehabilitation Hospital Affiliated to Fujian University of Traditional Chinese Medicine, involving 193 patients with flatfoot admitted from January 2021 to August 2025, including 144 surgically treated patients with flatfoot and AIS.

#### Phase 1

Risk Factor Analysis Cohort A total of 193 consecutive patients diagnosed with flexible flatfoot were enrolled for the initial risk factor analysis. This cohort included both patients with and without comorbid AIS. Inclusion Criteria for Phase 1 (all 193 patients) were: (1) Diagnosed with flexible flatfoot (absence of medial longitudinal arch upon weight-bearing); (2) Bilateral hallux valgus angle (HVA) and Meary's angle (MA) measurements available from weight-bearing radiographs; (3) (4) Normal hearing, speaking, reading, and writing abilities; (5) Provided signed informed consent by both adolescents and their guardians. Exclusion Criteria for Phase 1 were: (1) Psychiatric disorders or cognitive impairments affecting communication; (2) Severe comorbidities (e.g., cerebral palsy, neuromuscular diseases); (3) Previous spinal or major lower limb surgery.

Based on the presence of AIS (Cobb angle >10°), the 193 patients were categorized into the AIS group (*n* = 144) and the Non-AIS group (*n* = 49) for the comparative risk factor analysis.

#### Phase 2

Randomized Controlled Trial (RCT) Cohort From the Phase 1 cohort, the 144 patients diagnosed with both flatfoot and AIS who were scheduled for posterior corrective surgery (PCS) comprised the RCT population. Additional Inclusion Criteria for Phase 2 (RCT, *n* = 144) were: (1) Scheduled to undergo spinal correction, bone grafting fusion, and internal fixation (PCS); (2) No contraindications to anesthesia; (3) No history of allergies to medications used in the study protocol. Additional Exclusion Criteria for Phase 2 were: (1) Other conditions causing nausea or vomiting (e.g., gastrointestinal disorders); (2) Any condition deemed to make participation in the RCT unsafe by the surgical team.

These 144 patients were then randomly assigned to the Control group (routine care, *n* = 72) or the Comfort Nursing (CN) group (*n* = 72). The randomization procedure is detailed below.

*a priori* sample size calculation for the logistic regression was performed using G Power software (version 3.1). With an effect size (odds ratio) of 2.0, an alpha error of 0.05, and a power of 0.80, a minimum total sample of 170 was required. We recruited 193 participants to account for potential dropouts. For the RCT, a sample size of 64 per group was calculated to detect a 30% absolute reduction in PONV incidence (from 50% to 20%) with 80% power and a 5% significance level. We enrolled 72 per group to enhance statistical power.

The random allocation sequence was generated by an independent statistician using a computer-based random number generator in a 1:1 ratio. This sequence was implemented using sequentially numbered, opaque, sealed envelopes (SNOSE) to ensure allocation concealment. Upon a patient's written consent to participate, a research nurse, who was not involved in the study's outcome assessment, opened the next envelope in the sequence to reveal the group assignment (Control or CN). Due to the nature of the nursing intervention, blinding of the patients and the nursing staff administering the care was not feasible. However, outcome assessors (the orthopedic residents measuring foot parameters and the researchers collecting the PONV and questionnaire data) and the data analyst were blinded to the group allocation throughout the study period. No stratification or blocking was used in this study.

### Diagnostic criteria

The diagnosis of both conditions was confirmed using standardized weight-bearing radiographs and established clinical criteria. Flatfoot was determined by the International Classification of Diseases, Ninth Revision, Clinical Modification (ICD-9-CM) major primary diagnosis code 734 (Acquired flatfoot) and 754.61 (Congenital pes planus) (28). AIS was diagnosed based on standing posteroanterior (PA) full-spine radiographs. The diagnosis required a Cobb angle of ≥10°, measured by the standard technique, in a patient aged 10–18 years (29). All other known causes of scoliosis (e.g., congenital, neuromuscular) were excluded following clinical and radiographic evaluation by an experienced orthopedic surgeon.

### Foot parameter analysis

Foot parameters were measured by two experienced orthopedic residents, with averages calculated from spinal and foot imaging measurements. Foot parameters were measured on standardized weight-bearing anteroposterior and lateral radiographs of the foot using the hospital's picture archiving and communication system software. Measurement positions included: (1) HVA: the angle between the central axis of the first metatarsal and the proximal phalanx; (2) MA: the angle between the central axis of the talus (midpoint of the highest and lowest points connecting the talar body and head) and the long axis of the first metatarsal (midpoint of the distal and proximal ends); (3) *Δ*HVA: the difference in HVA between both feet, reflecting bilateral foot valgus symmetry; (4) *Δ*MA: the difference in MA between both feet, reflecting bilateral foot arch symmetry.

### Clinical approach

All patients undergoing surgery received identical surgical and anesthesia protocols. In the Control group, patients received standard nursing procedures, including education on anesthesia and surgery, postoperative analgesia side effects and management, and detailed guidance on P-NV prevention. Dietary education instructed patients to fast 6 h before surgery, with a repeat 4 h prior, and to gradually transition from liquids to regular food 6 h postoperatively.

For the CN group, patients underwent a series of comfort nursing interventions, including: (1) Preoperative individualized psychological nursing: assessing patients’ and families’ needs, providing tailored psychological counseling, using relaxation techniques to alleviate anxiety and reduce P-NV risk. Families were briefed on surgery and encouraged to maintain positive emotions; (2) Preoperative dietary nursing: recommending light and digestible foods preoperatively, transitioning to carbohydrate drinks or energy mixtures before fasting. Postoperatively, patients were encouraged to sip warm water, progressing to bland foods after 4 h, then semi-liquid diets. (3) Postoperative monitoring, positioning, and acupoint care: monitoring vital signs, adjusting bed angles (10°–30°) to alleviate pain and reduce P-NV. Axial turning and 30°–45°oblique lateral positioning were recommended. Acupoint massage (Neiguan and Hegu) and abdominal massage/heating were taught to promote gut motility. (4) Ensuring appropriate environments and clothing: maintaining optimal room temperature, ventilation, noise, and lighting levels. Providing comfortable bedding and clothing. (5) Emotional support: establishing rapport, offering emotional support, and promoting relaxation through communication, activities, and entertainment.

### Observational indicators

Patient demographics, N&V incidence (WHO criteria), VAS pain scores, P-NV symptoms (nausea, vomiting, dizziness, headache, anorexia, bloating, discomfort), and recovery (time, diet, nutrition, gastrointestinal function) were recorded. The incidence of PONV within the first 48 h postoperatively, assessed using the simplified PONV intensity scale (0 = no nausea/vomiting; I = mild nausea; II = severe nausea or single vomiting episode; III = two or more vomiting episodes; IV = intractable vomiting requiring rescue antiemetics). Pain intensity was assessed using the ​Visual Analog Scale (VAS, 0–10 cm) at baseline (preoperative). Postoperative vital signs (temperature, blood pressure, heart rate, urine output, respiratory rate) were recorded at 48 h after surgery. Post-discharge quality of life was assessed using a standardized scale, with higher scores indicating better quality.

### Statistical analysis

Data were analyzed using SPSS. The normal distribution of all continuous variables was tested using the Shapiro–Wilk test. Normally distributed continuous variables were described as mean ± standard deviation (SD) and analyzed using Independent Samples Student's *t*-test (normal distribution). Non-normally distributed continuous variables were described as median (interquartile range) and analyzed using Mann–Whitney *U* test. Categorical variables were described as numbers (n) and percentages (%) and analyzed using chi-square tests. Median and interquartile ranges were used for ordinal data, with Mann–Whitney *U* tests. *P* < 0.05 indicated statistical significance.

## Results

### Comparative analysis of foot parameters in patients with flatfoot

A total of 193 flatfoot patients were categorized into the AIS group (*n* = 144) or the Non-AIS group (*n* = 49) based on their scoliosis status. Patients in the AIS group were significantly older than those in the Non-AIS group (*P* < 0.001). The male-to-female ratio also differed significantly between the AIS (50/94) and Non-AIS (31/18) groups (*P* < 0.001). While no significant differences were found in the left or right foot Hallux Valgus Angle (HVA) and Meary's Angle (MA) between groups (*P* > 0.05), the asymmetry values (*Δ*HVA and *Δ*MA) were significantly higher in the AIS group (both *P* < 0.001, Table 1).

**Table 1:** Comparison of foot morphology parameters between flatfoot patients with and without AIS.

Baseline information	AIS group (*n* = 144)	Non-AIS group (*n* = 49)	t/Z/*X*^2^	*P*
Age (years)	16 (14,17)	12 (12,13)	Z = 4.217	<0.001
Gender (Male/Female)	50/94	31/18	*X*^2^ = 12.230	<0.001
Left HVA (°)	14.97 (12.16, 17.57)	15.01 (11.48, 17.50)	Z = 0.733	0.656
Right HVA (°)	14.62 ± 4.48	14.11 ± 3.79	t = 0.718	0.474
*Δ*HVA (°)	2.93 (1.54, 4.16)	1.30 (0.82, 1.89)	Z = 2.999	<0.001
Left MA (°)	14.39 (11.91, 17.58)	13.32 (11.21, 16.77)	Z = 0.815	0.520
Right MA (°)	14.52 (12.02, 17.62)	13.35 (11.13, 17.46)	Z = 0.736	0.651
*Δ*MA (°)	1.35 (0.69, 1.93)	0.32 (0.22, 0.56)	Z = 4.157	<0.001

### Risk factor analysis for AIS in patients with flatfoot

Univariate logistic regression identified age, gender, *Δ*HVA, and *Δ*MA as significant risk factors for AIS (Table 2). However, in the multivariate analysis adjusted for these variables, ​gender was not an independent risk factor​ (Table 3). Furthermore, multivariate Logistic regression analysis showed that age, *Δ*HVA, and *Δ*MA were independent risk factors for AIS. For every 1-unit increase in age, *Δ*HVA, or *Δ*MA, the risk of AIS in patients with flatfoot independently increased by 2.782, 7.277, or 3,806.31 times, respectively.

**Table 2:** Univariate logistic regression analysis for AIS in flatfoot patients.

Variable	B	SE	Wald*X*^2^	*P*	OR	95%CI
Age	0.910	0.135	45.411	<0.001	2.486	(1.907, 3.239)
Gender = Female	1.175	0.344	11.653	<0.001	3.238	(1.649, 6.356)
Left HVA	0.025	0.049	0.264	0.607	1.026	(0.931, 1.130)
Right HVA	0.028	0.039	0.518	0.472	1.028	(0.953, 1.110)
*Δ*HVA	1.123	0.208	29.038	<0.001	3.074	(2.043, 4.625)
Left MA	0.044	0.051	0.728	0.394	1.045	(0.945, 1.155)
Right MA	0.044	0.048	0.841	0.359	1.045	(0.951, 1.149)
*Δ*MA	5.749	1.059	29.462	<0.001	313.792	(39.366, 2,501.296)

**Table 3:** Multivariate logistic regression analysis for AIS in flatfoot patients.

Variable	B	SE	Wald*X*^2^	*P*	OR	95%CI
Age	1.330	0.376	12.495	<0.001	3.782	(1.809, 7.908)
Gender = Female	0.594	0.981	0.367	0.545	1.811	(0.265, 12.375)
Left HVA	−0.641	0.423	2.299	0.129	0.527	(0.230, 1.206)
Right HVA	0.483	0.359	1.808	0.179	1.621	(0.802, 3.278)
*Δ*HVA	2.113	0.653	10.479	0.001	8.277	(2.302, 29.759)
Left MA	−0.053	1.228	0.002	0.966	0.949	(0.086, 10.522)
Right MA	−0.145	1.177	0.015	0.902	0.865	(0.086, 8.693)
*Δ*MA	8.244	2.839	8.431	0.004	3,806.301	(14.577, 993,897.460)

### General data analysis of patients with flatfoot and AIS

The 144 AIS patients were randomized into the Comfort Nursing (CN) or Control group (*n* = 72 each). No significant differences were observed in baseline characteristics such as age, gender, BMI, operation time, or hospital stay between the two groups (all *P* > 0.05, Table 4), indicating successful randomization and group comparability.

**Table 4:** Baseline demographic and clinical characteristics of the flatfoot and AIS patients randomized to the control and CN groups.

Baseline information	Comfort nursing (*n* = 72)	Control (*n* = 72)	t/Z/*X*^2^	*P*
Age (years)	16 (14, 17)	16 (15, 17)	Z = 0.583	0.886
Gender (Male/Female)	21/51	29/43	1.961	0.161
BMI (kg/m^2^)	18.01 (16.30, 19.04)	18.01 (17.19, 19.25)	Z = 1.167	0.131
Operative time(hours)	3.24 ± 0.50	3.11 ± 0.57	t = 1.566	0.119
Hospital stay(days)	8.04 ± 1.05	8.16 ± 0.97	t = −0.736	0.463

### Incidence of N&V in patients

**Figure 1:**
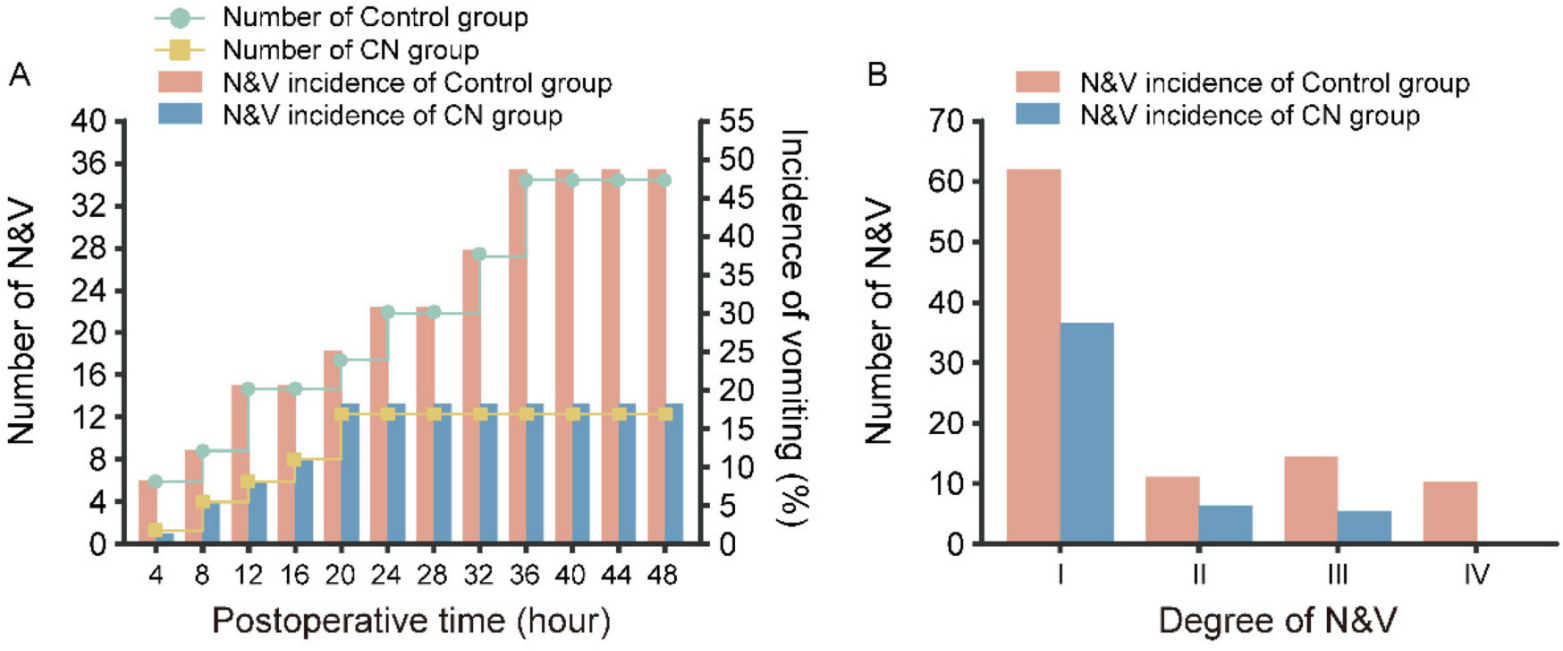
The Incidence of N&V in Patients with Flatfoot combined with AIS. **(A)** The Number and Incidence of N&V and **(B)** the Degree of N&V in CN group and Control group.

The incidence and severity of N&V in patients are shown in Figure 1. Within 48 h postoperatively, 35 cases (48.61%) of N&V occurred in the Control group, with 11, 14, and 10 patients experiencing grades II, III, and IV N&V, respectively. In contrast, 13 cases (18.06%) of N&V occurred in the CN group, with 7, 6, and 0 patients experiencing grades II, III, and IV N&V, respectively, significantly lower than the Control group (*P* < 0.05).

### Frequency of P-NV and pain intensity

The frequency of N&V was determined from nursing records, and pain intensity was assessed using the Visual Analog Scale (VAS). As shown in Table 5, the frequency of N&V was significantly higher in the Control group compared to the CN group (*P* < 0.001). Preoperative VAS scores were similar between groups (*P* > 0.05). Post-nursing, both groups showed reduced VAS scores, but the CN group had significantly lower scores than the Control group (*P* < 0.001), indicating that comfort nursing provided better pain relief than routine nursing.

**Table 5:** Frequency of postoperative nausea and vomiting (PONV) episodes and pain scores (VAS) in the control and CN groups.

Variable	CN group (*n* = 72)	Control group (*n* = 72)	Z/*X*^2^	*P*
N&V frequency (times)				
0	59 (81.94%)	37 (51.39%)	*X*^2^ = 21.391	<0.001
1	5 (6.94%)	4 (5.56%)		
2	5 (6.94%)	10 (13.89%)		
3	3 (4.17%)	11 (15.28%)		
4	0 (0.00%)	10 (13.89%)		
VAS before treatment	3.74 (2.89, 4.47)	3.60 (2.52, 4.37)	Z = 0.750	0.627
VAS after treatment	1.26 (1.13, 1.43)	2.60 (2.22, 3.06)	Z = 6.000	<0.001

### Postoperative vital signs in both groups

Postoperative vital signs, including body temperature, blood pressure, urine output, heart rate, and respiratory rate, are presented in Table 6. Body temperatures were 36.21 (36.08, 36.30)°C and 36.96 (36.55, 37.40)°C, systolic blood pressures were 99.80 ± 7.65 mmHg and 102.05 ± 7.51 mmHg, diastolic blood pressures were 73.36 ± 4.75 mmHg and 74.59 ± 6.03 mmHg, heart rates were 71 (66, 74) bpm and 72 (68, 75) bpm, urine outputs were 28.42 ± 7.04 mL/h and 25.02 ± 8.96 mL/h, and respiratory rates were 16 (13, 18) bpm and 15 (13, 17) bpm for the CN and Control groups, respectively. Statistically significant differences were observed in body temperature and urine output between the two groups (both *P* < 0.05).

**Table 6:** Comparison of postoperative physical characteristics.

Physical signs	CN group (*n* = 72)	Control group (*n* = 72)	t/Z	*P*
Temperature(°C)	36.21 (36.08, 36.30)	36.96 (36.55, 37.40)	Z = 4.583	<0.001
Systolic blood pressure (mmHg)	99.80 ± 7.65	102.05 ± 7.51	t = −1.780	0.077
Diastolic blood pressure (mmHg)	73.36 ± 4.75	74.59 ± 6.03	t = −1.363	0.175
Heart rate (bpm)	71 (66, 74)	72 (68, 75)	Z = 0.750	0.627
Urine output (mL/h)	28.42 ± 7.04	25.02 ± 8.96	t = 2.534	0.012
Respiratory rate(bpm)	16 (13, 18)	15 (13, 17)	Z = 0.917	0.370

### P-NV-related symptoms and medication use

**Figure 2:**
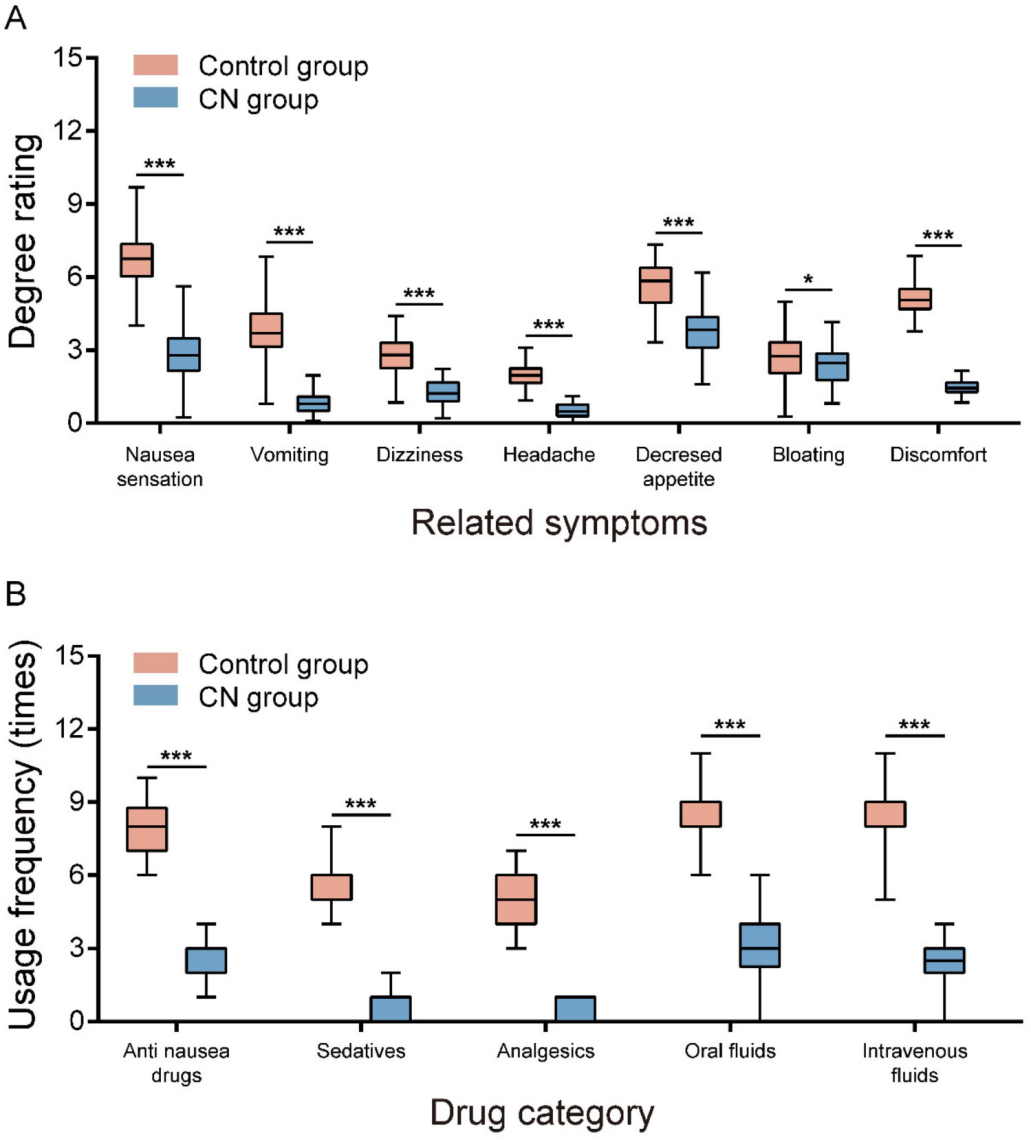
Comparison of N&V related symptoms and medication use between two groups of patients. **(A)** The degree rating of related symptoms and **(B)** Usage frequency of Drug in CN group and Control group.

As shown in Figure 2, P-NV-related symptoms in patients with flatfoot and AIS included nausea, vomiting, dizziness, headache, anorexia, bloating, and discomfort. Significant differences in symptom scores were observed between the two groups (*P* < 0.05). Medication for P-NV in AIS patients undergoing PCS was determined based on individual patient conditions and physician orders, including antiemetics, sedatives, analgesics, and oral or intravenous fluids. The CN group had significantly lower medication frequencies for all medications compared to the Control group (all *P* < 0.001).

### Postoperative recovery in both groups

Postoperative recovery and gastrointestinal function were evaluated based on recovery time, dietary intake, and nutritional status. As shown in Table 7, the CN group had significantly shorter recovery times than the Control group (*P* < 0.001). Additionally, the CN group had significantly better dietary intake and nutritional status than the Control group (both *P* < 0.001).

**Table 7:** Postoperative recovery and nutritional Status in both groups.

Physical signs	CN group (*n* = 72)	Control group (*n* = 72)	t/Z	*P*
Recovery time (days)	10.26 ± 3.52	13.37 ± 4.47	t = −4.633	<0.001
Dietary intake (kcal/day)	1,894.78 (1,862.35, 1,918.69)	1,495.14 (1,477.34, 1,526.45)	Z = 6.000	<0.001
Body weight gain (kg)	1.97 (1.35, 2.62)	1.19 (0.89, 1.51)	Z = 2.833	<0.001
Blood protein (g/L)	65.37 ± 5.74	57.89 ± 5.12	t = 8.243	<0.001
Blood vitamins (R/T)	8 (7, 9)	7 (5, 8)	Z = 2.167	<0.001
Blood electrolytes (R/T)	7 (6, 8)	5 (4, 7)	Z = 2.083	<0.001

In blood vitamin and electrolyte statistics, R/T represents the proportion of normal test items to the total number of test items.

### Patient satisfaction with nursing and post-discharge quality of life

Patient satisfaction with nursing was categorized as very satisfied, satisfied, neutral, or dissatisfied. As shown in Table 8, 59 patients in the Control group were satisfied, 9 were neutral, and 4 were dissatisfied, resulting in an. In contrast, the CN group had 29 very satisfied patients, 39 satisfied patients, 4 neutral patients, and 0 dissatisfied patients, resulting in a 94.44% satisfaction rate. The CN group had significantly higher patient satisfaction than the Control group. Quality of life was assessed using a standardized scale across seven domains: physical functioning, role-emotional, bodily pain, mental health, sleep quality, anxiety/depression, and general health. As shown in Table 7, the CN group had significantly higher post-discharge quality of life scores than the Control group in all domains (all *P* < 0.05).

**Table 8:** Post-Discharge patient satisfaction and quality of life scores in the CN and control groups.

QOL situation	CN group (*n* = 72)	Control group (*n* = 72)	t/Z/*X*^2^	*P*
Nursing satisfaction				
Very satisfied	29 (40.28%)	0 (0.00%)	*X*^2^ = 39.005	<0.001
Satisfied	39 (54.17%)	59 (81.94%)		
General level	4 (5.56%)	9 (12.50%)		
Dissatisfied	0 (0.00%)	4 (5.56%)		
Physiological activities	79.37 (74.03, 82.58)	69.62 (64.89, 74.00)	Z = 3.250	<0.001
Emotional awareness	86.653 ± 4.45	75.07 ± 5.12	t = 14.477	<0.001
Physical discomfort	86.39 ± 6.09	75.45 ± 6.03	t = 10.826	<0.001
Mental health	77.33 ± 5.94	65.83 ± 4.99	t = 12.586	<0.001
Sleep quality	79.98 (77.49, 83.55)	65.46 (61.92, 70.19)	Z = 5.583	<0.001
Anxiety or depression	80.92 (76.31, 84.76)	69.59 (67.74, 71.38)	Z = 4.833	<0.001
Overall health	84.88 ± 6.04	69.60 ± 5.13	t = 16.358	<0.001

## Discussion

Flatfoot is the most prevalent foot deformity in children, leading to increased foot load, physical discomfort, and limited mobility (30). Factors contributing to its development include gender, age, weight, ligament laxity, and hypermobility (4). AIS, an idiopathic scoliosis occurring during adolescent growth, disproportionately affects females, accounting for up to 60%–80% of cases (15). In our study, AIS patients displayed bilateral foot arch asymmetry and bilateral hallux valgus angle asymmetry. Based on these observations, we hypothesize that long-term asymmetric plantar pressure distribution in AIS patients impacts bilateral foot morphology, resulting in bilateral hallux valgus angle and foot arch asymmetry. Concurrently, this bilateral foot asymmetry may also influence the onset and progression of AIS. Our findings indicate that bilateral hallux valgus angle asymmetry (*Δ*HVA) and bilateral foot arch asymmetry (*Δ*MA) are risk factors for flatfoot complicated by AIS, potentially offering a novel diagnostic and therapeutic perspective worthy of further investigation.

Surgical intervention, a crucial management approach for AIS, while effective, is often accompanied by significant postoperative challenges, notably nausea and vomiting (N&V) (19). Clinical reports consistently show a high incidence of N&V following scoliosis surgery, which not only exacerbates patient suffering but also precipitates various complications (31). For instance, one study found that the incidence of postoperative N&V in scoliosis patients ranged from 30% to 50%, severely impacting postoperative recovery and quality of life (32). Complications associated with N&V include, but are not limited to, aspiration pneumonia, dehydration, electrolyte imbalances, and wound bleeding, further burdening patients. Consequently, the prevention and management of postoperative N&V in AIS patients are paramount. In this study, we aimed to explore the role of comfort nursing in reducing the incidence and symptoms of postoperative N&V in patients with idiopathic scoliosis complicated by flatfoot. We randomly assigned 144 patients with flatfoot and AIS into a Control group and a CN group, with 72 patients in each. The Control group received routine nursing, while the CN group received comfort nursing. Our study found no significant differences in baseline characteristics between the CN and Control groups (*P* > 0.05), indicating their comparability, which is crucial for subsequent analyses of the effects of comfort nursing. During the 48 h postoperative observation period, we meticulously recorded and compared the incidence, grades, frequencies, and pain levels of N&V between the two groups. Additionally, we systematically assessed postoperative vital signs, symptoms, medication use, and overall recovery in both groups and surveyed patients’ satisfaction with nursing services. The implementation of comfort nursing focused on enhancing patients’ physiological and psychological comfort to reduce postoperative N&V. Through this controlled study design, we aimed to provide more scientific and effective nursing strategies for postoperative care in patients with flatfoot complicated by AIS, thereby improving their quality of life and facilitating rapid recovery.

To effectively prevent and reduce postoperative N&V, comfort nursing strategies have been widely adopted in clinical practice (33). With the advancement of medical research and accumulation of nursing experience, comfort nursing has demonstrated remarkable efficacy in preventing and treating postoperative N&V in AIS patients (34). Clinical studies have shown that comfort nursing positively impacts postoperative N&V reduction (35). Through rational diet and hydration management, thirst and hunger discomfort can be significantly mitigated, lowering the incidence of N&V (36). A study on scoliosis patients found that shortening preoperative fasting times and early postoperative hydration significantly reduced N&V occurrences and improved patient comfort (37). In the current study, monitoring of postoperative vital signs, including body temperature, blood pressure, urine output, heart rate, and respiratory rate, revealed significant differences in body temperature and urine output between the CN group and Control group (both *P* < 0.05). Furthermore, Rigorous postoperative monitoring of N&V incidence revealed that within 48 h, the Control group had an N&V incidence of 48.16% (35/72), whereas the CN group had a significantly lower incidence of 18.06% (13/72), with less severe symptoms (*P* < 0.05). This finding aligns with previous studies reporting reduced N&V incidence in experimental groups, suggesting that specific nursing measures may effectively mitigate N&V. While N&V incidence reflects the occurrence of symptoms, the frequency and severity of P-NV are more critical. Our data showed that the Control group had significantly higher N&V frequencies than the CN group (*P* < 0.001).

Adequate pain control is a crucial measure in preventing N&V (37). Pain stimuli can trigger N&V; thus, judicious selection of analgesic medications and utilization of analgesia pumps effectively alleviate pain and subsequently reduce N&V risk (36). In this study, VAS scores indicated that pain was alleviated in both groups, but the CN group experienced a more pronounced reduction. Postoperative P-NV symptoms in patients with flatfoot and AIS, including nausea, vomiting, dizziness, and bloating, were significantly less severe in the CN group (*P* < 0.05), consistent with previous studies highlighting the effectiveness of specific nursing interventions in reducing pain and related symptoms (23). Moreover, with decreased N&V incidence and severity, the CN group required significantly fewer P-NV medications (*P* < 0.001), emphasizing the advantage of specific nursing measures in reducing medication use. Furthermore, postoperative sedation and psychological support positively influence patients’ anxiety and fear, contributing to a decrease in N&V episodes (38). In clinical practice, comfort nursing strategies have proven effective in preventing and reducing postoperative N&V (23). Our research findings also confirm this. postoperative recovery was significantly better in the CN group than in the Control group (all *P* < 0.001). Correspondingly, patient satisfaction [94.44% (68/72) *vs.* 81.94% (59/72)] and quality of life scores were also significantly higher in the CN group (both *P* < 0.001). Previous studies have also demonstrated the efficacy of specific nursing measures in enhancing patient satisfaction and quality of life (23). We anticipate that further research and exploration will optimize nursing measures, providing more personalized and precise care plans for patients. For instance, nursing plans tailored to individual differences and psychological characteristics, enhanced nursing effects and efficiency through advanced nursing technologies and equipment, and strengthened collaboration with other medical teams to form multidisciplinary comprehensive treatment models, will likely improve postoperative care for AIS patients and facilitate their comprehensive recovery.

While our study has confirmed the key effectiveness of comfort nursing in addressing postoperative N&V in AIS patients, several limitations persist. Firstly, the relatively small sample size may limit the generalizability of our findings; future studies should consider expanding the sample size to enhance the reliability of conclusions. Secondly, our study was conducted in a specialized rehabilitation hospital, potentially introducing regional limitations that affect the broader applicability of our results. Additionally, while our study demonstrates the efficacy of comfort nursing, its specific mechanisms remain incompletely understood; future research should delve into its physiological, psychological, and behavioral pathways to provide more scientific guidance for clinical practice. Furthermore, larger-scale randomized controlled clinical trials are warranted to further validate our findings and provide stronger evidence support. Finally, PONV, pain, symptom scores, and QoL were subjective and unblinded and may have been at risk of bias.

This study has several diagnostic limitations that should be considered. First, the assessment of risk factors (*Δ*HVA and *Δ*MA) was based on a retrospective analysis of a pre-existing flatfoot cohort. While this design efficiently identifies associations, it inherently limits our ability to establish a definitive causal relationship between foot asymmetry and the development of AIS. A prospective longitudinal study would be required to confirm causality. Second, although we demonstrated excellent inter-rater reliability, the radiographic measurements (HVA and MA) are subject to inherent variability based on patient positioning, technician technique, and the precision of the digital measurement tools. Future studies could employ more advanced 3D imaging modalities (e.g., weight-bearing CT) to obtain more precise and dynamic morphological data. Finally, while we adjusted for key confounders, the possibility of residual confounding from unmeasured variables (e.g., specific genetic markers, detailed gait analysis parameters, or muscle strength asymmetries) cannot be entirely ruled out. These factors might also contribute to the development of AIS and should be investigated in future research.

## Conclusion

In summary, our findings highlight the significant advantages of specific nursing measures, such as comfort nursing, in reducing postoperative nausea and vomiting (N&V) incidence, alleviating pain, promoting recovery, improving patient satisfaction, and enhancing post-discharge quality of life in patients with flatfoot complicated by idiopathic scoliosis. These results align with existing literature, further corroborating the effectiveness and importance of comfort nursing in postoperative care for AIS patients. This promising approach may offer a valuable strategy for improving clinical outcomes in comparable specialized rehabilitation settings. Future multi-center trials are warranted to confirm its efficacy and broader generalizability.

## Data Availability

The original contributions presented in the study are included in the article/Supplementary Material, further inquiries can be directed to the corresponding author.
